# Genetic Analysis of Egg Production Traits in Luhua Chickens: Insights from a Multi-Trait Animal Model and a Genome-Wide Association Study

**DOI:** 10.3390/genes15060796

**Published:** 2024-06-17

**Authors:** Qianwen Yang, Xubin Lu, Guohui Li, Huiyong Zhang, Chenghao Zhou, Jianmei Yin, Wei Han, Haiming Yang

**Affiliations:** 1 College of Mathematical Science, Yangzhou University, Yangzhou 225009, China; yzdxyqw@163.com; 2 College of Animal Science and Technology, Yangzhou University, Yangzhou 225009, China; lxb@yzu.edu.cn (X.L.); hmyang@yzu.edu.cn (H.Y.); 3 Jiangsu Institute of Poultry Science, Yangzhou 225611, China; sahui2008@163.com (G.L.); zhyong1983@163.com (H.Z.); reddsbhj3@163.com (C.Z.); 15952566122@163.com (J.Y.)

**Keywords:** egg production, genetic correlation, heritability, GWAS, gene ontology analysis, Luhua chicken

## Abstract

Egg production plays a pivotal role in the economic viability of hens. To analyze the genetic rules of egg production, a total of 3151 Luhua chickens were selected, the egg production traits including egg weight at first laying (Start-EW), egg weight at 43 weeks (EW-43), egg number at 43 weeks (EN-43), and total egg number (EN-All) were recorded. Then, the effects of related factors on egg production traits were explored, using a multi-trait animal model for genetic parameter estimation and a genome-wide association study (GWAS). The results showed that body weight at first egg (BWFE), body weight at 43 weeks (BW-43), age at first egg (AFE), and seasons had significant effects on the egg production traits. Start-EW and EW-43 had moderate heritability of 0.30 and 0.21, while EN-43 and EN-All had low heritability of 0.13 and 0.16, respectively. Start-EW exhibited a robust positive correlation with EW-43, while Start-EW was negatively correlated with EN-43 and EN-All. Furthermore, gene ontology (GO) results indicated that Annexin A2 (ANXA2) and Frizzled family receptor 7 (FZD7) related to EW-43, Cyclin D1 (CCND1) and A2B adenosine receptor (ADORA2B) related to EN-All, and have been found to be mainly involved in metabolism and growth processes, and deserve more attention and further study. This study contributes to accelerating genetic progress in improving low heritability egg production traits in layers, especially in Luhua chickens.

## 1. Introduction

Egg production not only holds significant economic importance for hens but also serves as a crucial indicator of breeders’ income. Laying performance can be assessed by various indexes, including egg number (EN), egg weight (EW), etc. EN provides insight into individual egg production within a specific timeframe and can aid in the selection of hens with higher laying performance [1]. EW holds significance for both egg producers and consumers [2]. Previous studies have indicated that EN is influenced by factors such as age at first egg (AFE) and correlates with body weight (BW) [3,4]. The season also affects egg production [5]. The peak in hens’ egg production occurs at the onset of the dry season, while it reaches its nadir towards the end of the rainy season [6].

Genetic parameters are mainly applied for analyzing and predicting the variation of quantitative traits and qualitative traits, serving as crucial references for the animal breeding process. EN has been estimated and ranged from 0.10 to 0.32 [7], suggesting the genetic basis of EN varied considerably among different breeds. de Freitas et al. found that the genetic correlation between AFE and total egg production of the D‘D’ layer line was −0.52, while Niknafs et al. found that for Mazandaran native chickens, it was only −0.41 [8,9]. These results indicated that there were large differences in the genetic basis for different breeds and genetic parameters were difficult to apply across breeds. Most of the egg-laying traits are of low heritability and hard to effectively improve in a short period of time by conventional breeding methods. In delving into the aforementioned challenges, the genome-wide association study (GWAS) has emerged as a prominent approach. It is recognized for its efficacy in identifying quantitative trait loci (QTLs) and genomic regions linked to egg-laying performance, as documented in previous studies [10,11]. This method holds promise for enhancing egg-laying traits through marker-assisted selection. Furthermore, numerous studies have been conducted on the genetic basis of egg production traits, revealing a plethora of genes or markers associated with EW and EN [1,12,13,14].

Luhua chickens, a domestic breed native to China, are renowned for their exceptional egg-laying performance, premium meat quality, and visually appealing appearance in both culinary and butchery contexts [15]. Recognized as a dual-purpose breed, it holds a crucial position as a valuable genetic resource within the poultry industry. Moreover, it is essential to harness its positive attributes for the development of top-tier chicken breeds. In this study, we comprehensively analyzed the effects of the BW, AFE, and season on the egg-laying performance of Luhua chickens and GWAS was utilized to identify candidate genes associated with EW and EN. Moreover, enrichment analyses were performed, which may be beneficial for the genetic improvement of egg-laying performance in hens.

## 2. Materials and Methods

### 2.1. Animals and Management

All animal experiments were conducted in accordance with the Institutional Animal Care and Use Committee of Yang Zhou University, Yang Zhou, China (No. SYXK(Su)2021-0020).

### 2.2. Chicken Population and Phenotypic Data

A total of 3151 healthy Luhua chickens were raised in the National Gene Bank of Local Chicken Breeds (Jiangsu, China), under the same housing conditions and diets fed. The housing environment was consistently maintained at a temperature of 20 °C, with a photoperiod of 16 h of light per day. Furthermore, the chickens were fed three times daily and had ad libitum access to both feed and water at all times. The production data for 3 generations (2020–2022) of Luhua chickens were recorded, each generation consisted of 80 families, and 905, 1066, and 1180 individuals, respectively. Egg weight at first laying (Start-EW), egg weight at 43 weeks (EW-43), egg number at 43 weeks (EN-43), total egg number (EN-All), body weight at first egg (BWFE), AFE, and body weight at 43 weeks (BW-43) were measured and recorded. Eggs from chickens of 3 generations were collected from 15 October 2020 to 23 June 2021, 20 October 2021 to 30 June 2022, and 20 November 2022 to 30 June 2023, respectively. More experimental grouping details of the tested chickens were shown in Table 1.

### 2.3. Phenotypic Data Analysis

Phenotypic data of four age groups were analyzed using R software (v. 4.3.2) to estimate the means, standard deviations (SD), and 95% confidence intervals for each trait separately. Phenotypic correlations among the traits were computed by Pearson’s method. Then, based on the correlation results, a normality test and ANOVA were performed on BWFE, AFE, BW-43, and season by the R function Shapiro Test and AoV, respectively.

### 2.4. Estimated Genetic Parameters

The genetic parameter estimation of the four egg production traits, which include Start-EW, EW-43, EN-43, and EN-All, were estimated by a multi-trait animal model with the DMU (v. 6-R5-2) software, and the average information restricted maximum likelihood (AI-REML) was used to estimate the (co)variance components. The multi-trait animal model was as follows:

(1) $$\left[\begin{array}{c} y_{1}\\ y_{2}\\ y_{3}\\ y_{4} \end{array}\right]=\left[\begin{array}{cccc} X_{1} & 0 & 0 & 0\\ 0 & X_{2} & 0 & 0\\ 0 & 0 & X_{3} & 0\\ 0 & 0 & 0 & X_{4} \end{array}\right]\left[\begin{array}{c} b_{1}\\ b_{2}\\ b_{3}\\ b_{4} \end{array}\right]+\left[\begin{array}{cccc} Z_{1} & 0 & 0 & 0\\ 0 & Z_{2} & 0 & 0\\ 0 & 0 & Z_{3} & 0\\ 0 & 0 & 0 & Z_{4} \end{array}\right]\left[\begin{array}{c} g_{1}\\ g_{2}\\ g_{3}\\ g_{4} \end{array}\right]+\left[\begin{array}{c} e_{1}\\ e_{2}\\ e_{3}\\ e_{4} \end{array}\right],$$

where *y_i_* (*i* = 1,2,3,4) is the vector of the 4 trait observations; *b_i_* is the vector of fixed effects, including BWFE, AFE, BW-43, and season; *g_i_* is the vector of additive genetic effects; ***e****_i_* is the random residuals; and **X** and **Z** are the association matrices, where **Z** is the kinship matrix, derived from generation information. The model was required for convergence at the criterion that the norm of the update vector was less than 1.0 × 10^−7^ or the norm of the gradient vector (AI) was less than 1.0 × 10^−6^.

Heritability and genetic correlations were calculated based on the estimated variance component as follows:

(2) $$h^{2}=\frac{\sigma_{a}^{2}}{\sigma_{a}^{2}+\sigma_{e}^{2}},\;r_{ij}=\frac{Cov(a_{i},a_{j})}{\sqrt{\sigma_{i}^{2}\sigma_{j}^{2}}},$$

where $h^{2}$ is the heritability of traits, $r_{ij}$ is the genetic correlation value between trait *i* and trait *j*,$\sigma_{a}^{2}$ is the additive genetic variance, $\sigma_{e}^{2}$ is the residual variance, and $Cov(a_{i},a_{j})$ is genetic covariance between trait *i* and trait *j*.

### 2.5. Genotypic Data and Quality Control

In this study, blood samples were collected randomly at 300 days of age and DNA was extracted from 60 Luhua chickens by a TIANamp Blood DNA Kit (TIANGEN BIOTECH (BEIJING) CO., LTD., Beijing, China, Cat. no: 4992208). After examining the DNA quality by polymerase chain reaction (PCR), a genome resequencing with a 10× sequencing depth was performed on the Illumina second-generation sequencing platform, and the assembled genome of chicken GRCg7b (GCF,016699485.2) was used as the reference genome. The single nucleotide polymorphisms (SNPs) were detected by GATK software (v. 4.4.0.0), and a total of 1,628,925 SNPs were detected. Then, SNPs were deemed unqualified if (1) the minor allele frequency (MAF) of an SNP was less than 0.05, (2) the call rate of an individual genotype was less than 99%, and (3) the SNP call rate was less than 90%. Finally, there remained 1,607,248 SNPs that were considered eligible for the following analyses.

### 2.6. Principal Component Analysis

To determine the level of population stratification, a principal component analysis (PCA) was conducted based on the 60 Luhua chickens’ 1,607,248 SNPs using the Plink software (v. 1.90), and a PCA plot was drawn using the ggplot2 package in R software (v. 4.3.2).

### 2.7. Association Analysis

GWAS was conducted by a mixed linear model for Start-EW, EW-43, EN-43, and EN-All independently in GEMMA (v. 0.98.5). The model was as follows:

***Y*** = ***Xb*** + ***Zu*** + ***e*** (3)

where ***Y*** is the vector of phenotype values; ***b*** is the vector of fixed effects, including BWFE, AFE, and BW-43; ***X*** is the incidence matrices of fixed effect vectors; ***u*** is the additive effect vector; ***Z*** is the incidence matrices of random effect vectors; and ***e*** is the random residual. Then, the CMplot package in R software (v. 4.3.2) was used to analyze the Manhattan plots of the GWAS. The Bonferroni test was used to reduce the occurrence rate of false positives.

### 2.8. Identification of Candidate Genes

To further explore the candidate genes, we identified genes within 200 kb based on the 200 SNPs with the smallest *p*-value for each of the four traits, respectively. Then, the above candidate genes were all submitted to the Cluster package in R software (v. 4.3.2) profiler to conduct the enrichment analysis, including GO analysis.

## 3. Results

### 3.1. Descriptive Statistics

Descriptive statistics of Start-EW, EW-43, EN-43, and EN-All of the different age groups are shown in Table 2. The Start-EW increased with AFE; however, EN-43 and EN-All both decreased with increasing AFE, and the mean values of EW-43 had minor change.

### 3.2. Correlation Analysis

**Figure 1:**
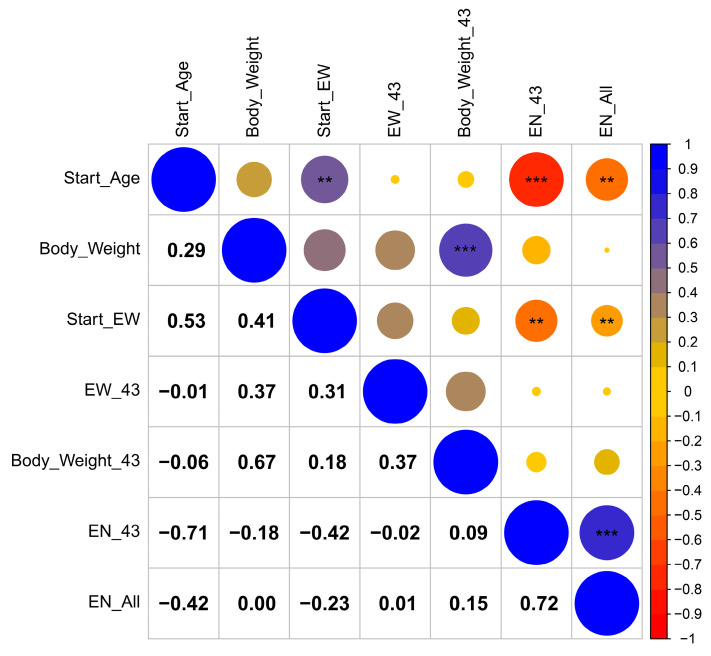
Phenotypic traits correlation between egg production traits. Egg production traits include age at first egg (Start_Age), body weight at first egg (Body_Weight), egg weight at first laying (Start_EW), egg weight at 43 weeks (EW_43), body weight at 43 weeks (Body_Weight_43), egg number at 43 weeks (EN-43), and total egg number (EN-All). The ** and *** represent significant correlations at 0.05 and 0.01, respectively.

A correlation analysis was performed between the four phenotypic traits (Figure 1); the results show that AFE is significantly positively correlated with Start-EW (r = 0.53, *p* < 0.05), while AFE is significantly negatively correlated with EN-43 (r = −0.71, *p* < 0.01) and EN-All (r = −0.42, *p* < 0.05). Moreover, Start-EW shows a moderately positive correlation with BWFE (r = 0.41), while Start-EW has a significant negative correlation with EN-43 (r = −0.42, *p* < 0.05) and EN-All (r = −0.23, *p* < 0.05).

### 3.3. Analysis of Variance

The results of ANOVA of BWFE, AFE, BW-43, and season are shown in Table 3, Table 4, Table 5 and Table 6, respectively. In Table 3, as AFE is delayed, Start-EW increases and EN-43 and EN-All gradually decrease, and there are significant differences among different groups (*p* < 0.05). Additionally, EW-43 in group 2 is significantly higher than in group 4. In Table 4, the number of individuals in each group of BWFE is 93, 732, 1143, 298, and 115, respectively, and ANOVA results reveal that both Start-EW and EW-43 increase with BWFE, while EN-43 decreases as BWFE increases (*p* < 0.05). For EN-All, there is no significant difference in the levels of BWFE among different groups (*p* > 0.05). In Table 5, BW-43 for each group is 331, 1635, 573, and 41. The Start-EW, EW-43, and EN-43 increase with BW-43, while EN-43 is significantly higher in group 1 than in other groups (*p* > 0.05). In Table 6, there is a high correlation between EN in winter and EN in spring (r = 0.597, *p* < 0.01), and a low correlation between EN in autumn and EN in winter and spring (r = 0.165, 0.094, *p* < 0.01). The results show that age, BWFE, and BW-43 have significant effects on the egg production traits. Therefore, we added the above three variables to fixed effects.

### 3.4. Genetic Parameter Statistics

The value of heritability, standard errors, and genetic correlations are shown in Table 7. The heritability is relatively higher for Start-EW, and lower for EW-43, EN-43, and EN-All. The standard errors of heritability estimates are small, suggesting that a reasonably high degree of reliance can be placed on these estimates. The results of the genetic correlations are shown in Table 8. Genetic correlations analyses reveal that there are significant positive genetic correlations between Start-EW and EW-43 (r = 0.52, *p* < 0.05), EN-43, and EN-All (r = 0.67, *p* < 0.05). In contrast, Start-EW and EW-43 are negatively correlated with EN-All.

### 3.5. Density Distribution of SNPs

**Figure 2:**
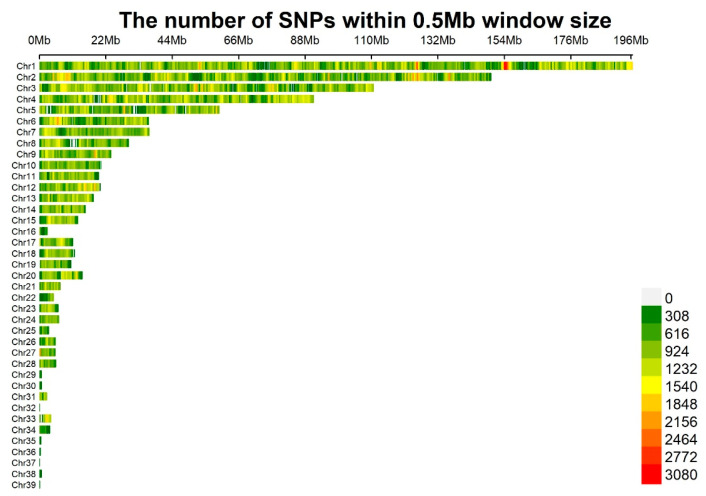
Density distribution of Single Nucleotide Polymorphisms (SNPs) on chromosomes. After conducting quality control, a total of 60 Luhua chickens and 1,607,248 SNPs remained. The distribution of the filtered SNPs is displayed over the 39 chromosomes.

In Figure 2, the SNP information is uniformly distributed on the chromosomes; moreover, the number of chromosomes detected is high in density in Chr1, within the range of 154–155.6 Mb, and there are some chromosomal deletions in Chr5 and Chr8, which may be caused by the sequencing technology. Since only a few of them could have a mutation, the impact on the following experiments could be neglected.

### 3.6. Principal Component Analysis

**Figure 3:**
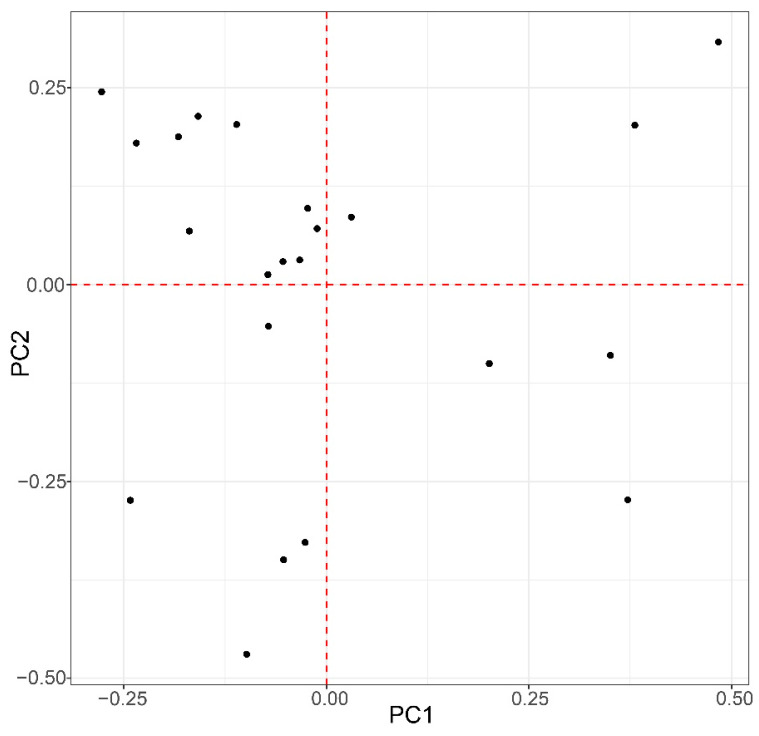
Population structure demonstrated by principal component analysis. Principal component analysis (PCA) was conducted with the 1,607,248 SNPs for the 60 Luhua chickens. The population structure is demonstrated by the scatter plots.

A PCA was used to visualize the population structure (Figure 3). The first three PCs exhibit 3.4%, 3.3%, and 2.8% of variation, respectively, which shows the existence of some population stratification.

### 3.7. Association Analysis

**Figure 4:**
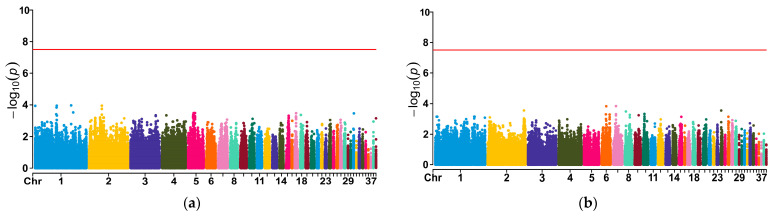

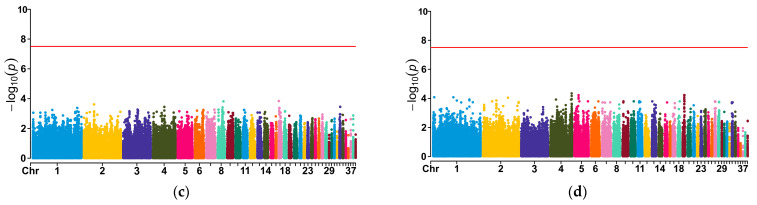
Manhattan plots of egg weight at first laying (**a**), egg weight at 43 weeks (**b**), egg number at 43 weeks (**c**) and total egg number (**d**). Manhattan plots established from the GWAS results of egg production traits in Luhua chickens. Manhattan plots display the negative logarithms of the observed *p* values for SNPs across 39 chormosomes.

Manhattan plots derived from the GWASs for the four traits are shown in Figure 4. However, no significant markers associated with the four egg production traits were found. The top 30 SNPs with the lowest *p*-value for each egg production trait are shown in the Supplementary Materials for further exploration of candidate genes (Tables S1–S4).

### 3.8. Enrichment Analysis

**Figure 5:**
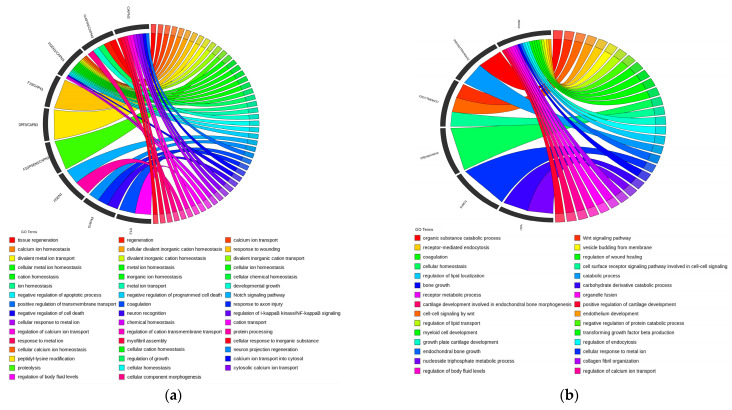

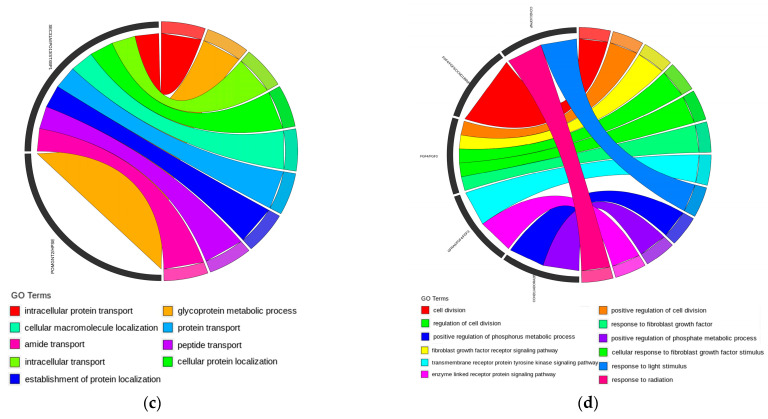
Gene ontology term results from egg weight at first laying traits (**a**), egg weight at 43 weeks traits (**b**), egg number at 43 weeks traits (**c**), and total egg number traits (**d**). GO analyses were conducted on the candidate genes with the smallest *p*-value by R package Cluster.

The results of an enrichment analysis (GO) for the four traits are shown in Figure 5. Figure 5 shows that differentially expressed genes (DEGs) of the top 30 SNPs affecting Start-EW are mainly enriched in tissue regeneration, regeneration, calcium ion transport, cellular calcium ion homeostasis, calcium ion homeostasis, and other aspects. DEGs of EW-43 are mainly enriched in organic substance catabolic processes, Wnt signalling pathways, cell–cell signaling by Wnt, endothelium development, and receptor-mediated endocytosis. DEGs of EN-43 are mainly enriched in intracellular protein transport, glycoprotein metabolic processes, intracellular transport, cellular protein localization, cellular macromolecule localization, and so on. Also, EN-All DEGs are mainly enriched in cell division, positive regulation of cell division, fibroblast growth factor receptor signaling pathways, cellular response to fibroblast growth factor stimulus, regulation of cell division, and other areas.

## 4. Discussion

### 4.1. Egg Production Analysis

Age at first egg, as the marker of the initiation of the reproductive period of the hens, is an extremely important indicator for reproduction [16].

In this study, the results showed that EN-43 and EN-All were significantly higher in group 1 than in other groups. Moreover, a negative correlation between age at first egg (AFE) and egg production was observed, suggesting that early sexual maturity may contribute to increased egg production [17]. This implies that selecting Luhua chickens with an earlier AFE, preferably 165 d or less, could be advantageous. Additionally, AFE exhibited various impacts on egg weight at different ages, particularly evident in the case of Start-EW, which demonstrated a strong positive correlation with AFE. An earlier AFE, smaller Start-EW, later AFE, and larger Start-EW, suggest that early sexual maturity consequently results in a reduction in EW [18,19]. Besides, an increase in late EW was relatively stable, and the weight of 43 egg weights were around 50 g, indicating a weak correlation with AFE.

With regards to BWFE, Start-EW and EW-43 were highest in group 5 and EN-43 in group 1, in agreement with the findings of Tongsiri et al., in which BWFE was positively correlated with Start-EW and negatively correlated with EW [20]. Start-EW and EW-43 were significantly higher in group 4 than in group 1. Wolc et al. and Noda et al. also found that body weight was moderately positively correlated with egg weight, suggesting that selecting for heavier BW-43 generally led to heavier egg weights [19,21]. 

For BW-43, EN-43 and EN-All were significantly lower in group 4 than in group 1, which was contrary to the findings of Begli et al., while similar to the findings of Lacin et al., which indicated that body weight traits (BW, BW-43) were weakly positively correlated with EN-ALL [22,23]. The results indicated that there were no significant genetic antagonisms between egg production and body weight (BW) among Luhua chickens. This suggests that an increase in the number of chickens with larger body weights in the flock could lead to an increase in both the weight and the number of eggs produced.

### 4.2. Genetic Evaluation

The heritability of Start-EW in Luhua chickens was 0.30, which was similar to Japanese quail (0.26) [24]. The heritability of EW43 in Luhua chickens was 0.21, lower than EW40 in the Giza M-2 line local broiler breeders (0.39–0.42), but higher than the Rhode Island red chicken (0.09) [25,26]. However, compared to most of other local breeds in China, the heritability of EW-43 in Luhua chickens was found to be slightly lower, which might be a common characteristic of the local breed; moreover, heritability seemed to decrease with age [27]. Meanwhile, there was a high genetic correlation between Start-EW and EW-43 (0.52), and a higher heritability for Start-EW (0.30) than for EW-43 (0.21), suggesting that the genetic selection of EW can be made early on the basis of the assessment of first egg weight.

The heritability of EN-43 in Luhua chickens was 0.13, which was in agreement with the findings of the Vanaraja female line chicken (0.15) and synthetic broiler dam line pullet (0.11) for EN 40, but lower than that of the broiler dam line (0.43) and Muscovy ducks (0.23) for EN40 [28,29,30,31]. The heritability of EN-ALL was 0.16, in agreement with the findings of Tongsiri et al. in Thai native chicken [19]. Similar to the findings of Savas et al., EN-43 showed high genetic correlation with EN-All (0.67), indicating its potential as a selection criterion for genetic improvement of egg production [32]. Furthermore, consistent with the results of Barot et al. [33], egg production was negatively genetically correlated with EW, suggesting that an excessive Start-EW would reduce EN to a certain extent. Therefore, when selecting for different traits, it is crucial to strike a balance among them, and carefully consider both the direction and intensity of selection in breeding lines. This comprehensive approach ensures that the main selected traits make progress while minimizing the impact on other traits.

### 4.3. Enrichment Analysis

In the case of the BW-43 DEGs of Start-EW, Calpain 3 (CAPN3) was abundantly expressed in the muscle of growing poultry, which was presumed to acted in development-related functions of muscle, such as control of skeletal cell proliferation or differentiation [34]. Growth-associated protein 43 (GAP43), known as neuromodulin, was involved in regulating the neuromast growth and actin cytoskeleton in neuronal cell lines, and acted as an integral part of the cortical cytoskeleton, influencing the cell surface weight and surface accumulation pattern of axonal growth cones [35,36]. Pre1senilin-1 (PSEN1), a part of γ-secretase, affected multiple processes, including amyloid precursor protein (APP) cleavage, β-calmodulin processing, Notch signaling, and calcium metabolism [37]. Double PHD fingers 3 (DPF3), presumed to be a major gene, expressed specifically in heart development, while it is also associated with skeletal muscle [38]. For differentially expressed genes (DEGs) associated with egg weight at 43 weeks (EW-43), Qiao et al. identified Annexin A2 (ANXA2). This gene is classified as a multifunctional calcium-dependent membrane phospholipid-binding protein, which plays various roles in regulating cellular functions. Its mRNA expression was found to be consistent with ovarian function and egg production in chickens. ANXA2 not only regulates its own expression but also participates in follicular angiogenesis, contributing to successful follicle development and ovulation [39,40]. Peroxiredoxin 6 (PRDX6), which is a bifunctional protein with glutathione peroxidase and calcium-independent phospholipase activity, played an important role in antioxidant defense [41,42]. Frizzled family receptor 7 (FZD7), a Wnt signaling receptor, was involved in the maintenance of mesenchymal phenotype, anorexia resistance, and spheroid and tumor formation in ovarian cancer (OC) [43]. Small ubiquitin-like modifier 1 (SUMO1), a member of the SUMO family, promoted protein homeostasis by affecting protein signaling or solubility, and cooperated, complemented, and balanced the ubiquitin-proteasome system at multiple levels [44]. 

Regarding the DEGs of EN-43, it was found that SEC31A could be used to regulate the collagen biosynthesis pathway and Sec31a upregulation would impair neuronal synapse growth [45]. Importin 13 (IPO13), a member of the importin-beta family of nuclear import proteins, transported cargoes into and out of the nucleus, and thus facilitated various important cellular processes [46]. β-1,4-N-acetylglucosaminyltransferase 2 (POMGNT2) could catalyze the construction of functional matrix glycan structures [47]. Heparanase protein (HPSE), a conserved protein in Aves, plays a significant role in cell membrane physiology. It is capable of disrupting the structural integrity of the basement membrane, leading to the release angiogenic and growth-promoting mediators [48]. 

For EN-All DEGs, Cyclin D1 (CCND1) was one of the intraovarian factors, which could furthermore influence hierarchical structure, folliculogenesis and follicular selection [49], and thus contribute to the improvement of egg production. Also, the findings of Mizushima et al. suggested that CCND1 was one of the key factors in the degradation of maternal retinoblastoma 1 proteins at the maternal-to-zygotic transition, contributing to cell cycle progression in subsequent follicular blastoderm development [50]. FGF3 and FGF4, members of FGF family, classified as the dysregulation of fibroblast growth factor, where FGF4 was associated with cellular proliferation and FGF3 worked in bone formation and limb development [51,52,53]. GDNF family receptor alpha 4 (GFRA4) might affect cell proliferation, cell cycle progression, apoptosis, and invasion, giving rise to neuroprotective effects in the enteric neural crest [54]. The A2B adenosine receptor (ADORA2B) played an essential role in the physiology of penile erection in smooth muscle cells [55]. 

## 5. Conclusions

A significant positive correlation was observed between Start-EW and EW-43, with both traits exhibiting relatively high heritability. Conversely, Start-EW was negatively correlated with EN-43 and EN-All, which both showed relatively low heritability. Additionally, the GO analysis revealed associations between egg production and certain genes, including ANXA2, FZD7, CCND1, and ADORA2B. These findings could offer valuable insights into understanding the genetic architecture of low heritability egg-laying traits in layers, particularly in Luhua chickens.

## Figures and Tables

**Table 1:** Experimental Grouping Details *.

Groups	AFE	BW-43	BWFE
1	147–165 d	1010–1380 g	1060–1270 g
2	166–183 d	1380–1750 g	1270–1480 g
3	184–201 d	1750–2120 g	1480–1690 g
4	202–220 d	2120–2490 g	1690–1890 g
5	/	/	1890–2100 g

* AFE, age at first egg (d); BW-43, body weight at 43 weeks (g); BWFE, body weight at first egg.

**Table 2:** Descriptive statistics for egg production traits *.

Traits	Age	*n*	Mean	SD	95% Confidence Intervals	
					Upper Limit	Lower Limit
Start-EW	1	569	35.22	0.16	34.92	35.53
	2	1235	37.80	0.11	37.59	38.01
	3	652	41.10	0.15	40.81	41.39
	4	125	42.27	0.33	41.61	42.92
EW-43	1	569	49.92	0.17	49.59	50.24
	2	1235	50.36	0.11	50.14	50.58
	3	652	49.93	0.16	49.63	50.24
	4	125	49.69	0.35	48.99	50.38
EN-43	1	569	129.81	0.61	128.62	131.00
	2	1235	116.53	0.41	115.72	117.34
	3	652	98.30	0.57	97.19	99.41
	4	125	83.13	1.30	80.59	85.67
EN-All	1	569	206.62	1.57	203.55	209.69
	2	1235	191.06	1.06	188.97	193.14
	3	652	166.68	1.46	163.81	169.55
	4	125	147.13	3.34	140.58	153.68

* Start-EW, egg weight at first laying (g); EW-43, egg weight at 43 weeks (g); EN-43, egg number at 43 weeks; EN-All, total egg number.

**Table 3:** Effects of age at first egg on egg production traits *.

Age at First Egg	*n*	Start-EW	EW-43	EN-43	EN-All
1	569	35.22 ± 0.15 ^d^	49.92 ± 0.17 ^ab^	129.81 ± 0.61 ^a^	206.62 ± 1.57 ^a^
2	1235	37.80 ± 0.11 ^c^	50.36 ± 0.11 ^a^	116.53 ± 0.41 ^b^	191.06 ± 1.06 ^b^
3	652	41.10 ± 0.15 ^b^	49.93 ± 0.16 ^ab^	98.30 ± 0.57 ^c^	166.68 ± 1.46 ^c^
4	125	42.27 ± 0.33 ^a^	49.69 ± 0.35 ^b^	83.13 ± 1.30 ^d^	147.13 ± 3.34 ^d^

* Start-EW, egg weight at first laying; EW-43, egg weight at 43 weeks; EN-43, egg number at 43 weeks; EN-All, total egg number. For each column of data, the upper subscripts with different letters indicate significant differences (*p* < 0.05).

**Table 4:** Effects of body weight at first egg on egg production traits *.

Body Weight at First Egg	*n*	Start-EW	EW-43	EN-43	EN-All
1	93	34.44 ± 0.41 ^e^	47.80 ± 0.38 ^e^	116.70 ± 1.98 ^a^	180.60 ± 4.23 ^a^
2	732	36.52 ± 0.15 ^d^	48.53 ± 0.14 ^d^	116.77 ± 0.71 ^a^	185.49 ± 1.51 ^a^
3	1143	38.41 ± 0.12 ^c^	50.27 ± 0.11 ^c^	113.53 ± 0.56 ^a^	187.66 ± 1.21 ^a^
4	498	40.32 ± 0.18 ^b^	51.79 ± 0.17 ^b^	108.80 ± 0.86 ^b^	185.52 ± 1.83 ^a^
5	115	42.44 ± 0.37 ^a^	53.47 ± 0.34 ^a^	104.19 ± 1.78 ^c^	183.74 ± 3.81 ^a^

* Start-EW, egg weight at first laying; EW-43, egg weight at 43 weeks; EN-43, egg number at 43 weeks; EN-All, total egg number. For each column of data, the upper subscripts with different letters indicate significant differences (*p* < 0.05).

**Table 5:** Effect of body weight at 43 weeks on egg production traits *.

Body Weight at 43 Weeks	*n*	Start-EW	EW-43	EN-43	EN-All
1	331	37.22 ± 0.24 ^c^	48.17 ± 0.20 ^d^	108.98 ± 1.06 ^b^	166.56 ± 2.20 ^b^
2	1635	38.13 ± 0.11 ^c^	49.72 ± 0.09 ^c^	113.31 ± 0.48 ^ab^	188.22 ± 0.99 ^a^
3	573	39.17 ± 0.18 ^b^	52.15 ± 0.16 ^b^	115.44 ± 0.81 ^a^	191.81 ± 1.67 ^a^
4	41	40.44 ± 0.67 ^a^	53.82 ± 0.58 ^a^	114.15 ± 3.01 ^a^	188.07 ± 6.26 ^a^

* Start-EW, egg weight at first laying; EW-43, egg weight at 43 weeks; EN-43, egg number at 43 weeks; EN-All, total egg number. For each column of data, the upper subscripts with different letters indicate significant differences (*p* < 0.05).

**Table 6:** Effect of seasons on egg number.

Item		Autumn	Winter	Spring
Autumn	Pearson Correlation	1	0.165 **	0.094 **
	Significance (two-tailed)		<0.001	<0.001
	*n*	2980	2980	2980
Winter	Pearson Correlation	0.165 **	1	0.597 **
	Significance (two-tailed)	<0.001		<0.001
	*n*	2980	3149	3149
Spring	Pearson Correlation	0.094 **	0.597 **	1
	Significance (two-tailed)	<0.001	<0.001	
	*n*	2980	3149	3149

** At the 0.01 level (two-tailed), the correlation was significant.

**Table 7:** Value of heritability and standard error *.

Traits	Heritability	Heritability Standard Error
Start-EW	0.299	0.049
EW-43	0.213	0.048
EN-43	0.132	0.040
EN-All	0.162	0.047

* Start-EW, egg weight at first laying; EW-43, egg weight at 43 weeks; EN-43, egg number at 43 weeks; EN-All, total egg number.

**Table 8:** Genetic correlations between egg production traits.

Item	Start-EW	EW-43	EN-43	EN-All
Start-EW	1.00	0.52	−0.31	−0.30
EW-43	0.52	1.00	−0.23	−0.15
EN-43	−0.31	−0.23	1.00	0.67
EN-All	−0.30	−0.15	0.67	1.00

## Data Availability

The original contributions presented in the study are included in the article/supplementary material, further inquiries can be directed to the corresponding author. Egg number per hen was recorded every day. Body weight and the weight of three consecutive eggs laid in the initial stage of production and 43 weeks were weighed. The venous blood was collected from wings at 300 days of age, and then the blood samples were sent to Shenzhen Huada Gene Technology Co., Ltd.

## Supplementary Materials

The following supporting information can be downloaded at: https://www.mdpi.com/article/10.3390/genes15060796/s1, Table S1: Top 30 significant SNPs in Start-EW. Table S2: Top 30 significant SNPs in EW-43. Table S3: Top 30 significant SNPs in EN-43. Table S4: Top 30 significant SNPs in EN-All.
