# Supplementary material for: Genetic Analysis of Egg Production Traits in Luhua Chickens: Insights from a Multi-Trait Animal Model and a Genome-Wide Association Study
Source: Genes (Basel). 2024 Jun 17;15(6):796. doi: 10.3390/genes15060796 (PMC11202424; doi:10.3390/genes15060796)
Supplement: Supplementary file 1 [file genes-15-00796-s001.zip › genes-3019721-supplementary.pdf]

Table S1. Top 30 significant SNPs in Start-EW *.

Chr	SNP	bp	A1	A2	b	se	p
1	NC_052532.1:137414986	137414986	A	G	-5.7012	1.4729	0.0001
2	NC_052533.1:49022583	49022583	T	C	5.7089	1.4785	0.0001
2	NC_052533.1:49022879	49022879	T	C	5.7089	1.4785	0.0001
2	NC_052533.1:49022949	49022949	G	A	5.7089	1.4785	0.0001
2	NC_052533.1:49023324	49023324	T	C	5.7089	1.4785	0.0001
1	NC_052532.1:82322947	82322947	C	T	-5.9460	1.5404	0.0001
1	NC_052532.1:82340429	82340429	T	C	-5.9460	1.5404	0.0001
1	NC_052532.1:146644	146644	A	T	-6.0514	1.5701	0.0001
1	NC_052532.1:81530550	81530550	T	C	-6.3057	1.6617	0.0001
2	NC_052533.1:48904212	48904212	T	C	5.3152	1.4189	0.0002
2	NC_052533.1:48905017	48905017	A	T	5.3152	1.4189	0.0002
1	NC_052532.1:145329239	145329239	G	C	-6.4675	1.7882	0.0003
5	NC_052536.1:26047213	26047213	C	A	-5.0516	1.4060	0.0003
5	NC_052536.1:25064039	25064039	T	C	-4.9498	1.3778	0.0003
5	NC_052536.1:25173689	25173689	T	G	-4.9498	1.3778	0.0003
5	NC_052536.1:25186247	25186247	G	A	-4.9498	1.3778	0.0003
5	NC_052536.1:25186250	25186250	C	T	-4.9498	1.3778	0.0003
5	NC_052536.1:25186325	25186325	C	T	-4.9498	1.3778	0.0003
5	NC_052536.1:25186336	25186336	T	A	-4.9498	1.3778	0.0003
5	NC_052536.1:25186337	25186337	A	C	-4.9498	1.3778	0.0003
5	NC_052536.1:25186344	25186344	C	T	-4.9498	1.3778	0.0003
5	NC_052536.1:25186538	25186538	A	G	-4.9498	1.3778	0.0003
5	NC_052536.1:25186547	25186547	G	T	-4.9498	1.3778	0.0003
5	NC_052536.1:25186717	25186717	C	G	-4.9498	1.3778	0.0003
5	NC_052536.1:25192577	25192577	C	T	-4.9498	1.3778	0.0003
5	NC_052536.1:25192933	25192933	A	T	-4.9498	1.3778	0.0003
5	NC_052536.1:25193001	25193001	T	C	-4.9498	1.3778	0.0003
5	NC_052536.1:25224758	25224758	A	G	-4.9498	1.3778	0.0003
5	NC_052536.1:25224797	25224797	A	C	-4.9498	1.3778	0.0003
5	NC_052536.1:25225248	25225248	T	A	-4.9498	1.3778	0.0003

* SNP, Single Nucleotide Polymorphism.

Table S2. Top 30 significant SNPs in EW-43.

Chr	SNP	bp	A1	A2	b	se	p
2	NC_052533.1:142898529	142898529	T	C	7.6129	2.0953	0.0003
1	NC_052532.1:152912411	152912411	A	G	6.9492	2.0522	0.0007
1	NC_052532.1:152925194	152925194	G	A	6.9492	2.0522	0.0007
1	NC_052532.1:152950544	152950544	G	A	6.9492	2.0522	0.0007
1	NC_052532.1:153185164	153185164	A	G	6.9492	2.0522	0.0007

1	NC_052532.1:153185371	153185371	G	A	6.9492	2.0522	0.0007
1	NC_052532.1:153188371	153188371	A	T	6.9492	2.0522	0.0007
1	NC_052532.1:153188758	153188758	T	G	6.9492	2.0522	0.0007
1	NC_052532.1:153189406	153189406	T	G	6.9492	2.0522	0.0007
1	NC_052532.1:153189484	153189484	C	T	6.9492	2.0522	0.0007
1	NC_052532.1:5021990	5021990	A	G	-8.5375	2.5230	0.0007
1	NC_052532.1:5556631	5556631	G	A	-8.5375	2.5230	0.0007
1	NC_052532.1:5577700	5577700	A	T	-8.5375	2.5230	0.0007
1	NC_052532.1:7645780	7645780	T	G	-8.5375	2.5230	0.0007
1	NC_052532.1:7773238	7773238	T	C	-8.5375	2.5230	0.0007
1	NC_052532.1:7773297	7773297	C	T	-8.5375	2.5230	0.0007
1	NC_052532.1:7773810	7773810	C	T	-8.5375	2.5230	0.0007
1	NC_052532.1:7773817	7773817	T	G	-8.5375	2.5230	0.0007
1	NC_052532.1:7773953	7773953	G	A	-8.5375	2.5230	0.0007
1	NC_052532.1:7774216	7774216	T	C	-8.5375	2.5230	0.0007
1	NC_052532.1:7774364	7774364	A	G	-8.5375	2.5230	0.0007
1	NC_052532.1:7774553	7774553	A	G	-8.5375	2.5230	0.0007
1	NC_052532.1:7836004	7836004	A	G	-8.5375	2.5230	0.0007
1	NC_052532.1:7839167	7839167	G	A	-8.5375	2.5230	0.0007
1	NC_052532.1:7872643	7872643	T	G	-8.5375	2.5230	0.0007
1	NC_052532.1:8044158	8044158	A	G	-8.5375	2.5230	0.0007
1	NC_052532.1:8044957	8044957	C	G	-8.5375	2.5230	0.0007
1	NC_052532.1:8045518	8045518	A	G	-8.5375	2.5230	0.0007
1	NC_052532.1:8045565	8045565	A	T	-8.5375	2.5230	0.0007
1	NC_052532.1:154744120	154744120	T	C	-8.5375	2.5230	0.0007

Table S3. Top 30 significant SNPs in EN-43.

Chr	SNP	bp	A1	A2	b	se	p
2	NC_052533.1:41114085	41114085	G	A	12.9371	3.5360	0.0003
4	NC_052535.1:45773207	45773207	C	A	12.6897	3.5640	0.0004
1	NC_052532.1:179205369	179205369	C	T	-12.2782	3.4904	0.0004
3	NC_052534.1:57817080	57817080	A	C	13.7328	3.9796	0.0006
1	NC_052532.1:78423368	78423368	G	A	13.6687	3.9796	0.0006
3	NC_052534.1:59449835	59449835	T	C	-11.9055	3.4851	0.0006
4	NC_052535.1:45664690	45664690	A	G	11.7211	3.4364	0.0006
4	NC_052535.1:45714969	45714969	G	A	11.7211	3.4364	0.0006
4	NC_052535.1:45719678	45719678	T	G	11.7211	3.4364	0.0006
4	NC_052535.1:45719894	45719894	A	G	11.7211	3.4364	0.0006
4	NC_052535.1:45760560	45760560	G	A	11.7211	3.4364	0.0006
4	NC_052535.1:45762698	45762698	A	G	11.7211	3.4364	0.0006
4	NC_052535.1:45764811	45764811	C	T	11.7211	3.4364	0.0006

4	NC_052535.1:45773036	45773036	G	A	11.7211	3.4364	0.0006
4	NC_052535.1:45773118	45773118	G	T	11.7211	3.4364	0.0006
4	NC_052535.1:45773188	45773188	C	A	11.7211	3.4364	0.0006
4	NC_052535.1:45773195	45773195	G	T	11.7211	3.4364	0.0006
4	NC_052535.1:45778691	45778691	G	T	11.7211	3.4364	0.0006
4	NC_052535.1:45780062	45780062	C	T	11.7211	3.4364	0.0006
4	NC_052535.1:45780162	45780162	T	C	11.7211	3.4364	0.0006
4	NC_052535.1:45780323	45780323	G	A	11.7211	3.4364	0.0006
4	NC_052535.1:45780385	45780385	T	G	11.7211	3.4364	0.0006
4	NC_052535.1:45782929	45782929	T	A	11.7211	3.4364	0.0006
4	NC_052535.1:45782954	45782954	C	T	11.7211	3.4364	0.0006
4	NC_052535.1:45783031	45783031	A	G	11.7211	3.4364	0.0006
4	NC_052535.1:45783055	45783055	A	G	11.7211	3.4364	0.0006
4	NC_052535.1:45783079	45783079	C	T	11.7211	3.4364	0.0006
4	NC_052535.1:45783183	45783183	G	A	11.7211	3.4364	0.0006
5	NC_052536.1:5948220	5948220	T	G	-10.9802	3.2494	0.0007
3	NC_052534.1:24799967	24799967	C	G	-13.6630	4.0436	0.0007

Table S4. Top 30 significant SNPs in EN-All.

Chr	SNP	bp	A1	A2	b	se	p
4	NC_052535.1:88784751	88784751	A	G	-49.5970	12.1906	0.00005
4	NC_052535.1:88784824	88784824	G	T	-49.5970	12.1906	0.00005
4	NC_052535.1:88791264	88791264	T	C	-49.5970	12.1906	0.00005
4	NC_052535.1:88828167	88828167	T	C	-49.5970	12.1906	0.00005
4	NC_052535.1:88828180	88828180	A	G	-49.5970	12.1906	0.00005
4	NC_052535.1:88828191	88828191	A	G	-49.5970	12.1906	0.00005
4	NC_052535.1:88845975	88845975	A	G	-49.5970	12.1906	0.00005
4	NC_052535.1:88846448	88846448	T	G	-49.5970	12.1906	0.00005
5	NC_052536.1:17075636	17075636	T	G	-48.4481	12.1000	0.00006
5	NC_052536.1:17077469	17077469	T	C	-48.4481	12.1000	0.00006
5	NC_052536.1:17077603	17077603	C	T	-48.4481	12.1000	0.00006
5	NC_052536.1:17078082	17078082	G	A	-48.4481	12.1000	0.00006
5	NC_052536.1:17084412	17084412	C	T	-48.4481	12.1000	0.00006
5	NC_052536.1:17084442	17084442	C	T	-48.4481	12.1000	0.00006
5	NC_052536.1:17085176	17085176	C	T	-48.4481	12.1000	0.00006
5	NC_052536.1:17086125	17086125	A	G	-48.4481	12.1000	0.00006
5	NC_052536.1:17086380	17086380	C	G	-48.4481	12.1000	0.00006
5	NC_052536.1:17088011	17088011	G	A	-48.4481	12.1000	0.00006
5	NC_052536.1:17088012	17088012	C	T	-48.4481	12.1000	0.00006
5	NC_052536.1:17088986	17088986	C	T	-48.4481	12.1000	0.00006
5	NC_052536.1:17088988	17088988	A	G	-48.4481	12.1000	0.00006

5	NC_052536.1:17089516	17089516	C	G	-48.4481	12.1000	0.00006
5	NC_052536.1:17089564	17089564	C	T	-48.4481	12.1000	0.00006
5	NC_052536.1:17099544	17099544	C	T	-48.4481	12.1000	0.00006
5	NC_052536.1:17100040	17100040	G	T	-48.4481	12.1000	0.00006
4	NC_052535.1:88941103	88941103	T	A	-54.0359	13.5992	0.00007
1	NC_052532.1:4359639	4359639	T	C	-53.0848	13.5282	0.00009
1	NC_052532.1:4360012	4360012	G	A	-53.0848	13.5282	0.00009
1	NC_052532.1:4360050	4360050	G	C	-53.0848	13.5282	0.00009
1	NC_052532.1:4360573	4360573	T	C	-53.0848	13.5282	0.00009
